# Antibacterial effects of nano-imprinted moth-eye film in practical settings

**DOI:** 10.1371/journal.pone.0198300

**Published:** 2018-10-03

**Authors:** Miho Yamada, Kiyoshi Minoura, Takashi Mizoguchi, Kenichiro Nakamatsu, Tokio Taguchi, Takuya Kameda, Miho Sekiguchi, Tatsuo Suzutani, Shinichi Konno

**Affiliations:** 1 Display Technology Development Center, Development Group, Display Device Company, Sharp Corporation, Tenri, Nara, Japan; 2 New Business Promotion Center, Development Group, Display Device Company, Sharp Corporation, Tenri, Nara, Japan; 3 Department of Orthopaedic Surgery, Fukushima Medical University School of Medicine, Fukushima, Japan; 4 Department of Microbiology, Fukushima Medical University School of Medicine, Fukushima, Japan; VIT University, INDIA

## Abstract

**Background:**

Recent studies report that surfaces displaying micrometer- or nanometer-sized undulating structures exhibit antibacterial effects. In previous work, we described the use of an advanced nanofabrication technique to generate an artificial biomimetic Moth-eye film by coating a polyethylene terephthalate (PET) film with nanoscale moth-eye protrusions made from a hydrophilic resin. This moth-eye film exhibited enhanced antibacterial effects in *in vitro* experiments. The aim of the present study was to verify the antibacterial efficacy of the Moth-eye film in practical environments.

**Materials and methods:**

The antibacterial effects of three types of film (Moth-eye film, Flat film, and PET film) were compared. Sample films were pasted onto hand washing basins at the testing locations. After several hours, bacteria were collected from the surface of the sample films with one of three kinds of culture media stamper (to permit identification of bacterial species). The stampers were incubated for 48 hours at 35°C, and the numbers of colonies were counted.

**Results and discussion:**

The number of common bacteria including *E*. *coli* and *S*. *aureus* obtained from the Moth-eye film was significantly lower than those from the PET film (p<0.05) and Flat film at 1 hour (p<0.05). This study found that the Moth-eye film showed a long-term (6h) antibacterial effect and the Moth-eye structure (PET coated with nanoscale cone-shaped pillars) demonstrated a physical antibacterial effect from earlier time points. Therefore, the Moth-eye film appears to have potential general-purpose applications in practical environments.

## Introduction

A large number of patients, ranging from 648,000 to 1,700,000 patients per year in the United States, suffer from healthcare-associated infections (HCAIs) [1]. In response to this situation, efforts are being made to better understand and control the spread of various infectious diseases. The prevention of hospital infections starts with hand washing[2]. Frequent sterilization and/or disinfection of environmental surfaces are also important. However, it is impossible to maintain hygienic cleanliness over an entire medical institution where bacterial carriers and the readily infected are expected to mix. The situation has become more serious than ever with the appearance of drug-resistant bacteria such as methicillin-resistant *Staphylococcus aureus* (MRSA), vancomycin-resistant *Enterococcus* (VRE), and multi-drug-resistant *Pseudomonas aeruginosa* (MDRP) [3, 4]. Antimicrobial technologies have been developed in response to the strong demand reliable and effective action against hospital infections. Further, the incorporation of antibacterial materials into advanced medical equipment, where such materials would need to exhibit high stability and safety, is also a target of recent research.

Various mechanisms for infection control have been widely used, such as chemical agents, exposure to high temperature, high pressure and ultraviolet rays, and incorporation of photocatalysts and so on. Silver particles [5] are among the more useful antibacterial agents. Materials that include silver particles are regarded as safe and durable, and can help prevent the growth of drug-resistant bacteria. Therefore, this material has been incorporated into a wide variety of environments, ranging from household products to medical equipment. Still, there are growing concerns as to whether the long-term exposure of bacteria to silver particles may actually provoke the development of drug-resistant bacteria, such as nanosilver-resistant microorganisms [6].

The spread of various drug-resistant pathogens, other than nanosilver-resistant pathogens, has prompted researchers to come up with novel antimicrobial systems that integrate a bio-inspired approach with nanotechnology[3, 7, 8]. These new approaches can be mainly classified into three types. The first is the utilization of nature-derived materials as antibacterial agents. For example, chitin and chitosan [9] extracted from crustacean shells, and natural plant-derived ingredients such as wasabi (Japanese horseradish) and mustard [10] are well-known for their potent antibacterial activities. The second is the synthesis of organic antibacterial agents such as eumelamine [11,12] and peptide [13], both of which are found in living organisms. They can be produced by the templated synthesis of inorganic nanoparticles. The synthesized agents mimic not only the chemical elemental ratio but also the molecular morphology and microstructure of those found in organisms. All of these unique properties are considered to be related to the observed antibacterial effects. The third is the utilization of surfaces displaying bio-inspired micrometer- or nanometer-sized undulations that exhibit physical antibacterial effects [7]. For instance, the wings of dragonflies and cicadas have been reported to have the ability to kill bacteria [14–16]. Shark skin [17] and sacred lotus leaf [18] have the ability to prevent the attachment of bacteria. These structures exhibit regular or random undulations ranging in size from several tens of nanometers to several micrometers. Artificial nanostructured surfaces having a regular array of pillars, approximately 200-nm high and spaced approximately 170 nm apart, have also been reported to have physical bactericidal effects [19].

We have developed an artificial moth-eye film that is fabricated using an advanced nano-imprinting technique. The resulting film possesses unique nanostructure arrays that exhibit a variety of useful functions, including super-hydrophilic or super-hydrophobic properties due to the larger surface area of the moth-eye film compared to a flat surface. We previously reported that our moth-eye film exhibited enhanced antibacterial effects *in vitro* [20]. However, the moth-eye film has not been evaluated for antibacterial effects in a practical setting. Hand wash basins are considered to be a major source of hospital infection [2] due to environmental causes and the use of the basins by large numbers of people. Therefore, the aim of the present study was to verify the antibacterial efficacy of the moth-eye film through its practical application to hospital hand wash basins.

## Materials and methods

The protocol for this study was approved by the Ethics Committee at Fukushima Medical University School of Medicine (IRB No.2829), representing the central ethics committee, and/or ethics committees/IRBs of the participating institutions.

### Materials

Three types of sample films were used: a polyethylene terephthalate (PET) film as a control; Moth-eye film, consisting of PET film coated with nanoscale cone-shaped pillars made of hydrophilic resin; and Flat film, consisting of PET film coated with a flat surface made of hydrophilic resin. The Moth-eye coating was fabricated by a nano-imprinting method using an ultra-violet curable resin and a Moth-eye stamper possessing an array of nanoscale cone-shaped holes [2]. Each single unit structure on the Moth-eye film was a cone-shaped protrusion that corresponded to the shape of the unit structure on the stamper; i.e., a hole of approximately 200 nm in depth and diameter. To generate the Flat film, the Moth-eye stamper was replaced with a flat glass stamper surface.

### Sample film lamination

Sample films, each consisting of a 4-cm-square section of the respective material, were pasted onto the vertical surface inside each basin and the horizontal surface around the edge of each basin. The arrangement of sample sections was changed for each test in order to prevent the number of bacterial species collected from being influenced by the sample location. After laminating, each of the sample film surfaces and each basin were disinfected by wiping three times with an ethanol-impregnated paper cloth, and that time was taken as the start time of each test.

### Bacteria collection

Culture media stampers smaller than the size of the sample film were used to collect bacteria. Each sample film was stamped by one stamper. The contact time of the stamper on the film was approximately three seconds. Three types of stampers were used: plate count agar (PCA) made of standard agar medium, mannitol salt with egg yolk agar (MSEY)[21], and *Escherichia coli* (ES) Colimark agar (ESCM) containing sodium lauryl sulfate[22] (Eiken Chemical Co. Ltd., Japan).

### Test locations

A total of 6 hand wash basins in three bathrooms (two basins in each bathroom) were chosen at Fukushima Medical University and its associated hospital. The total number of sample films was 108 (48 Moth-eye, 48 PET, and 12 Flat). Due to the limited basin area, only PCA stampers were used for all three types of films, with MSEY stampers and ESCM stampers only used for Moth-eye film and PET film. Detailed data sets are available at doi.org/10.6084/m9.figshare.6246419.v1. The basins were in constant use from 10:00 to 16:00, which correspond to the test time. The test was designed so that bacteria were collected at 1 hour and 6 hours after start time on each day of testing. After collection, the culture media stampers were incubated and the numbers of colonies were counted. Approximately 40 people and 10 people per hour used each bathroom at the hospital and university, respectively.

An additional test was conducted at the cafeteria in Sharp Corporation Tenri factory, as the numbers of samples were too low to reveal any statistically significant differences. A total of 4 hand wash basins were chosen at the company cafeteria. The number of sample films was 92 (46 Moth-eye, and 46 PET). The basins were used intensively during the factory lunch break, from 11:30 to 13:00. The start time for the test was 11:00, and bacteria were collected at 2 hours (13:00) after the start time on the day of testing. Following collection, the culture media stampers were incubated and numbers of colonies were counted. 550 people used the basins between 11:00 and 13:00.

To prevent any bias, the sampler who collected bacteria with stampers and the measurer who counted the numbers of colonies were assigned separately. Therefore, the measurer counted the colonies blinded to the detailed information on the sample films.

**Fig 1:**
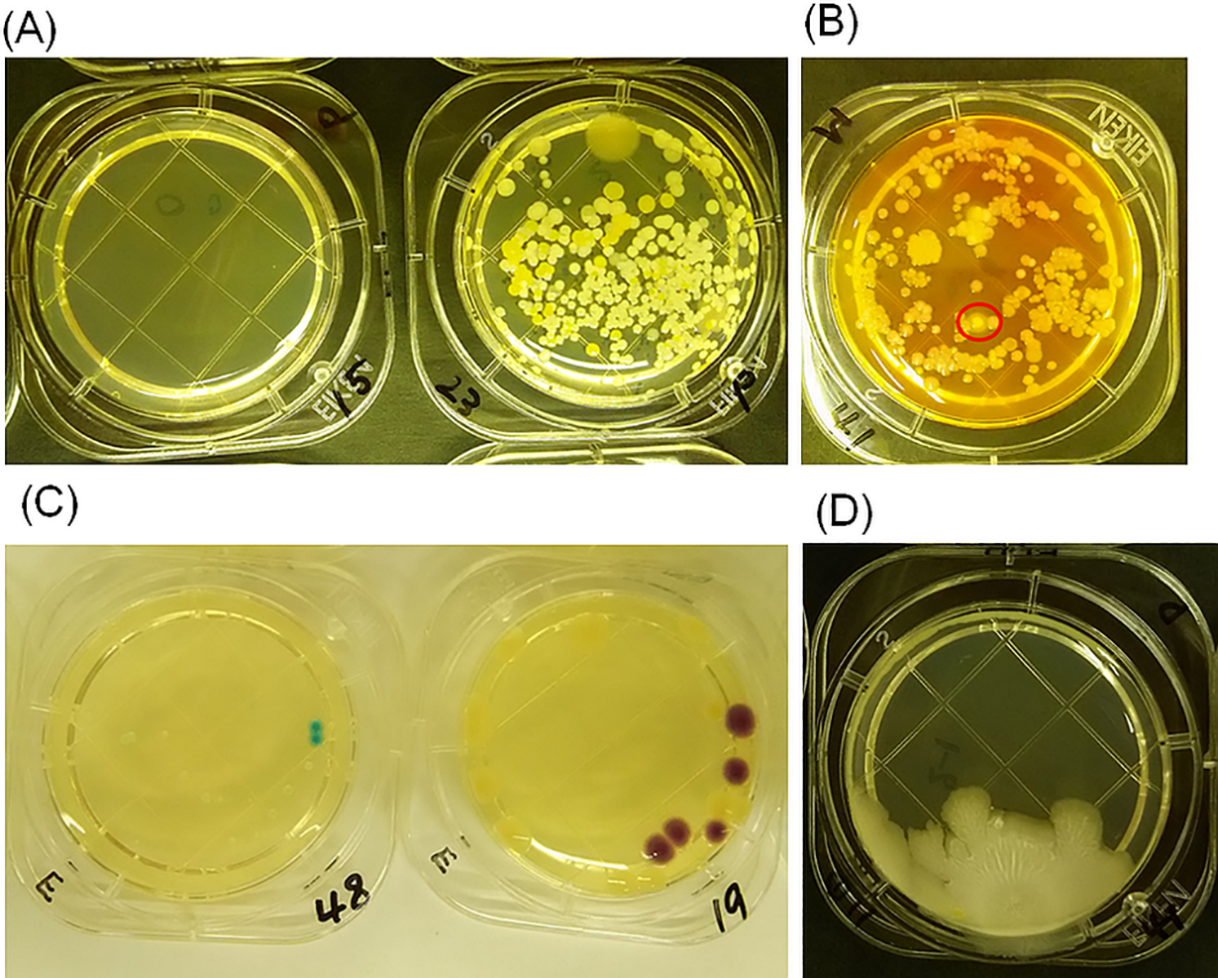
Identification of bacteria species and colonies on each agar. Various species of common bacteria, S. group and *S*. *aureus* bacteria, and *Escherichia coli* or coliform bacteria were identified on (A) PCA agar, (B) MSEY agar, and (C) ESCM agar respectively. Also, one sheet colony was identified on (D) PCA agar.

When counting, for all cases where the colonies were too numerous to count accurately, the number was defined as too-numerous-to-count (TNTC) and set at 400 for averaging purposes. Typical pictures of the PCA, MSEY and ESCM stampers after collecting bacteria are shown in Fig 1(A), 1(B) and 1(C), respectively. Sheet colonies that covered a relatively large area of the agar were omitted from the counts, and they were excluded from the evaluation of antibacterial efficacy. The reason for the exclusion was to avoid miscounting and ensure the evaluation can be quantified. In other words, the number of sheet colonies was not countable as each of them resembled a leaf and they partially overlapped with each other. A typical picture of the sheet colonies on a PCA stamper is shown in Fig 1(D).

### Statistical data processing

All data are reported as the mean and standard deviation. A two-tailed Mann-Whitney test was used to compare the effects of the individual films. A p-value less than 0.05 was considered statistically significant.

### Scanning electron microscope (SEM) observation

The collection of films for SEM observation was conducted at the company cafeteria. A total of 2 hand wash basins were chosen at the company cafeteria. The number of 2-cm-square sample films was 36 (12 Moth-eye, 12 PET, and 12 Flat). As with the bacteria collection, the start time was 11:00 and sample film collection was set at 16:00.

Sample films were fixed with 2.5% glutaraldehyde and paraformaldehyde for 1 hr to suppress chemical reactions due to bacterial death. The sample films were then dewatered with gradually increasing ethyl-alcohol concentrations and freeze-dried with tertiary-butyl alcohol repeatedly at ethanol concentrations of 60%, 70%, 80%, 90%, 95%, 100% and again 100% and by 4 times replacement from ethanol to tertiary-butyl alcohol in the same manner at concentrations of 30%, 50%, 70%, 100% and again at 100%. Lastly, the sample films were fixed on the observation base with conductive tape and a 5-nm osimium film was deposited by sputtering. Microscopic analysis was carried out with an S-4300SE field emission scanning electron microscope (Hitachi High-Technologies, Tokyo, Japan).

## Results and discussion

**Fig 2:**
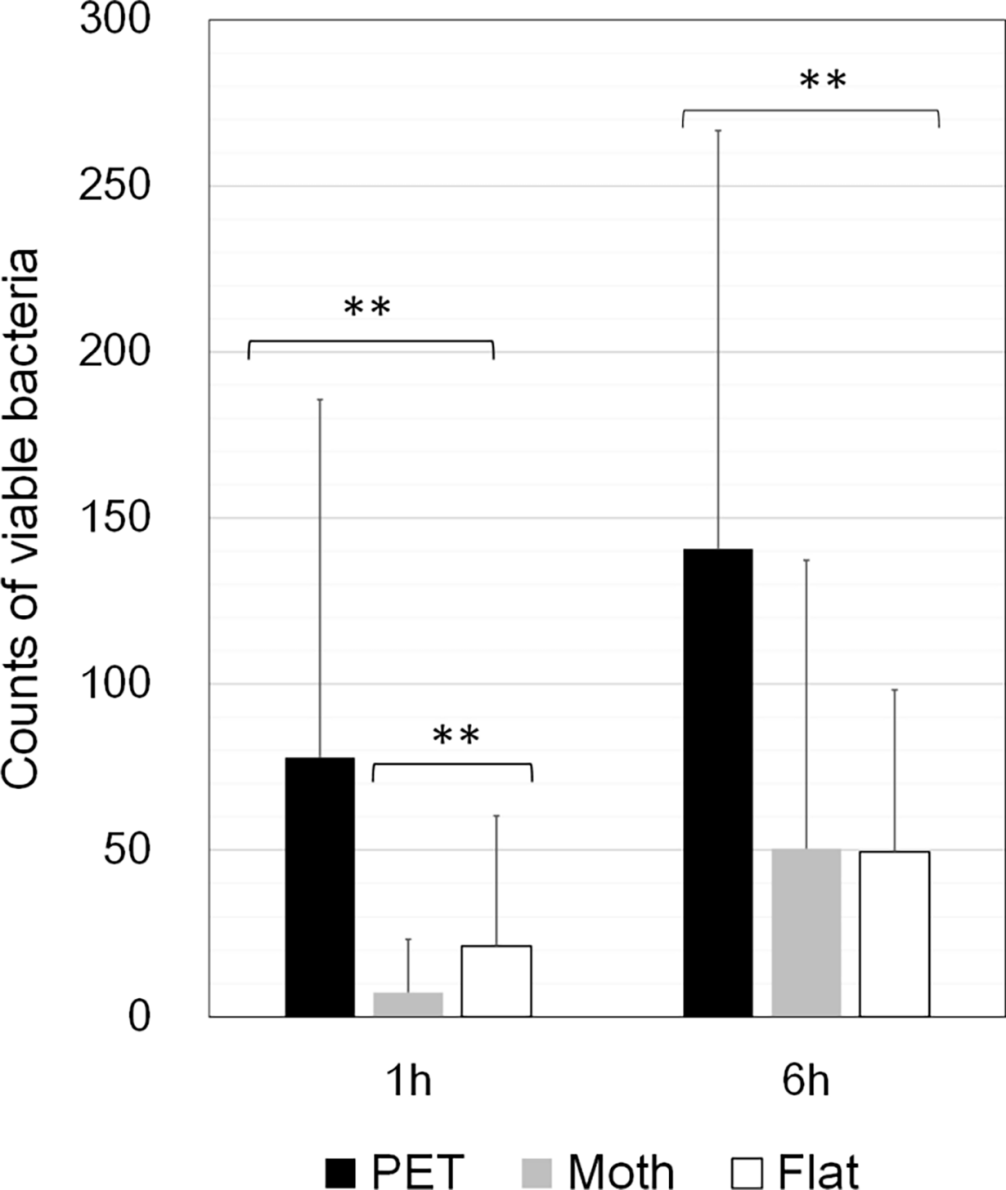
Common bacteria counts on the PCA stampers from the university hospital bathrooms. Average numbers of viable bacteria collected from the Moth-eye, Flat, and PET films at 1 and 6 hours. ** p<0.05, Mann-Whitney test. n = 24.

The mean counts of live bacteria collected by PCA stamper from the three films are shown in Fig 2. At both 1 and 6 hours, the mean counts of common bacteria collected from the Moth-eye and Flat films were significantly lower than those from the PET film (p<0.05). In comparison, those from Moth-eye film also were significantly lower than those from the Flat film at 1 hour (p<0.05) but not at 6 hours. These results indicate that the Moth-eye film has two interesting properties when applied in a practical setting; it is both immediately effective and shows a long-term (6 hours) antibacterial effect.

First, the antibacterial effect was provided by the chemical components in the resin, and the immediate antibacterial effectiveness of the Moth-eye film was considered enhanced by hydrophilic resin nanostructure, as was reported in our previous *in vitro* study [20]. Furthermore, the super-hydrophilic property of the Moth-eye film leads to quicker drying of water droplets adhering to the moth-eye surface. The nanostructures enhanced the intrinsic hydrophobic and hydrophilic properties of the resin. This phenomenon is well-known as Wenzel's law [23] as is based on the contact angle of a liquid droplet on a rough surface. This drying effect was expected to make it difficult for bacteria to grow on this surface. Nevertheless, no significant difference was found between the antibacterial effects of the Flat and the Moth-eye film surfaces at 6 hours. There are two possible reasons for the result. First, one set of TNTC data collected from the Moth-eye film showed a great effect, although the data was only one set out of the 24 data sets collected from Moth-eye film at 6 hours (see the detailed data at doi.org/10.6084/m9.figshare.6246419.v1). Second, both the Moth-eye film and the Flat film became completely wet at 6 hours, the completely wet state of the surface is considered to be the reason for the absence of any difference in the antibacterial effect as was mentioned in our prior work in the laboratory.

Second, it was confirmed that the antibacterial effect of the Moth-eye and Flat films coated with hydrophilic resin was retained for longer than that of the PET film. Alcohol, a well-known disinfective agent, has an immediate effect, but the effect is rapidly lost via its evaporation. It has been reported that the recovery of viruses and bacteria from stainless steel surfaces at 1 h after cleaning with ethanol ranged from 24 to 76% [24]. Most solid disinfective chemical agents exhibit their effects by diffusion in water; the antibacterial efficacy of these agents is lost once the agent is completely eluted. Efforts to provide longer effectiveness have included adsorbing silver ions onto zeolite [25] and maintaining high concentrations of silver ions by using a super-hydrophilic binder [26]. At the same time, chemical agents are known to have certain negative impacts or risks, notably via the killing of useful microbial organisms and via the evolution of drug-resistant bacteria.

**Fig 3:**
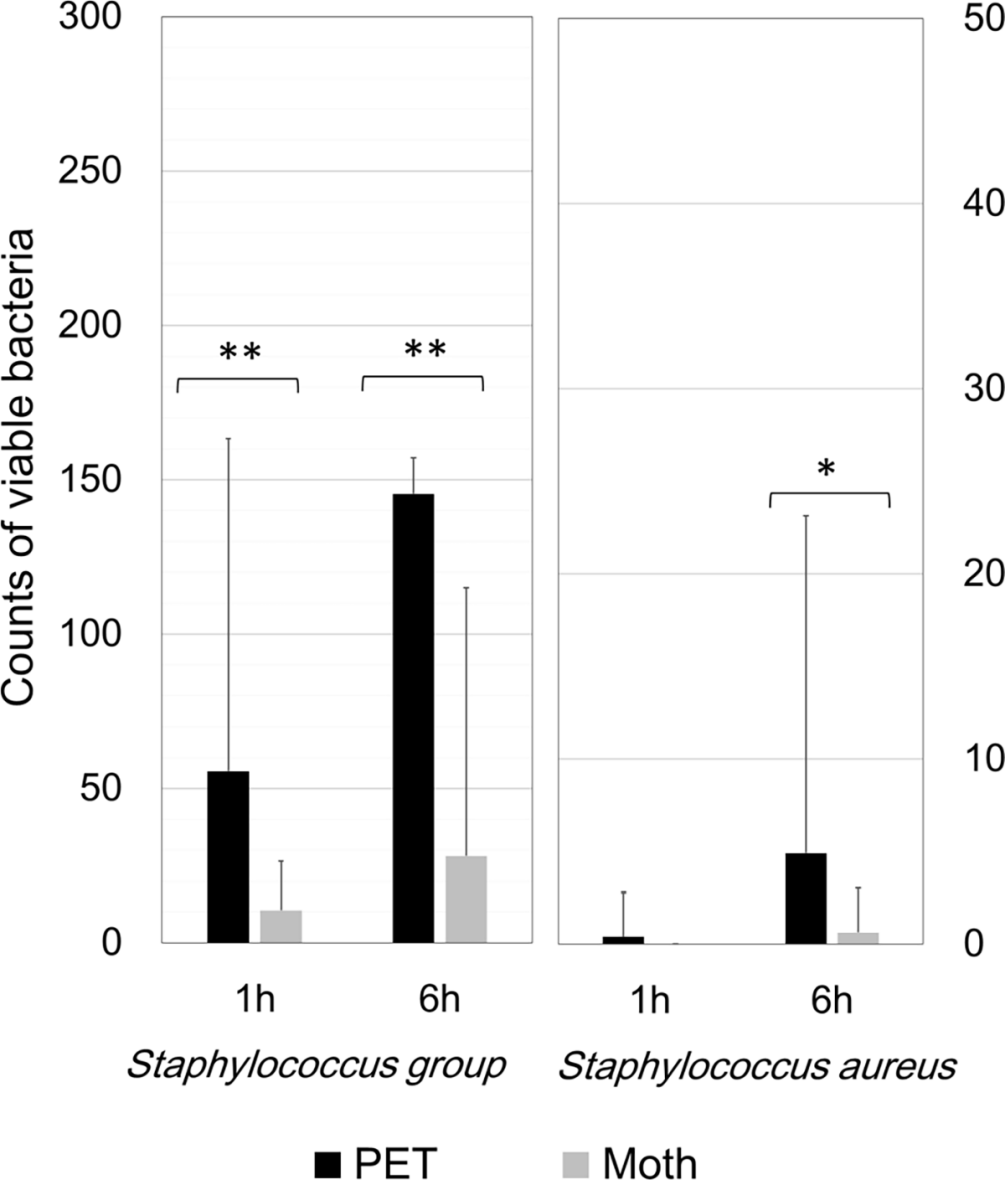
*Staphylococcus* species counts on the MSEY stampers from the university hospital bathrooms. Average numbers of viable bacteria, *Staphylococcus* species (a) and *Staphylococcus aureus* (b), collected from the Moth-eye and PET films at 1 and 6 hours. ** p<0.05, Mann-Whitney test. n = 40.

**Fig 4:**
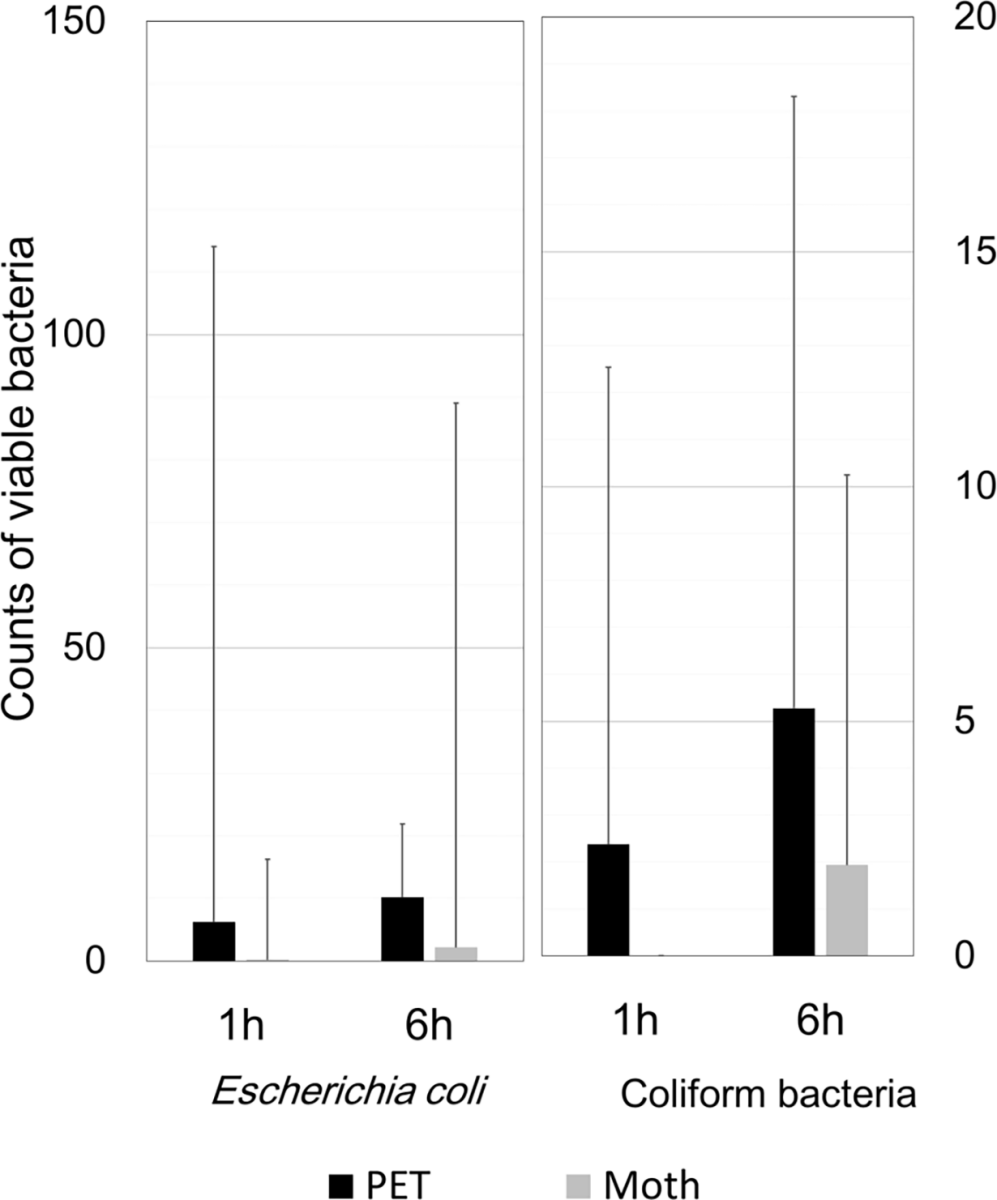
*Escherichia coli* and coliform bacteria counts on the ESCM stampers from the university hospital bathrooms. Average numbers of viable bacteria, *Escherichia coli* (a) or coliform bacteria (b), collected from the Moth-eye and PET films at 1 and 6 hours. The numbers of bacteria did not significantly differ between the two films at either time point. n = 32.

The mean counts of live bacteria collected by MSEY stamper from the Moth-eye and PET films are shown in Fig 3 and those collected by ESCM are shown in Fig 4. Regarding the *S*. species and *S*. *aureus*, the numbers of colonies collected from the Moth-eye film were significantly lower than those collected from PET film (p<0.05).

**Fig 5:**
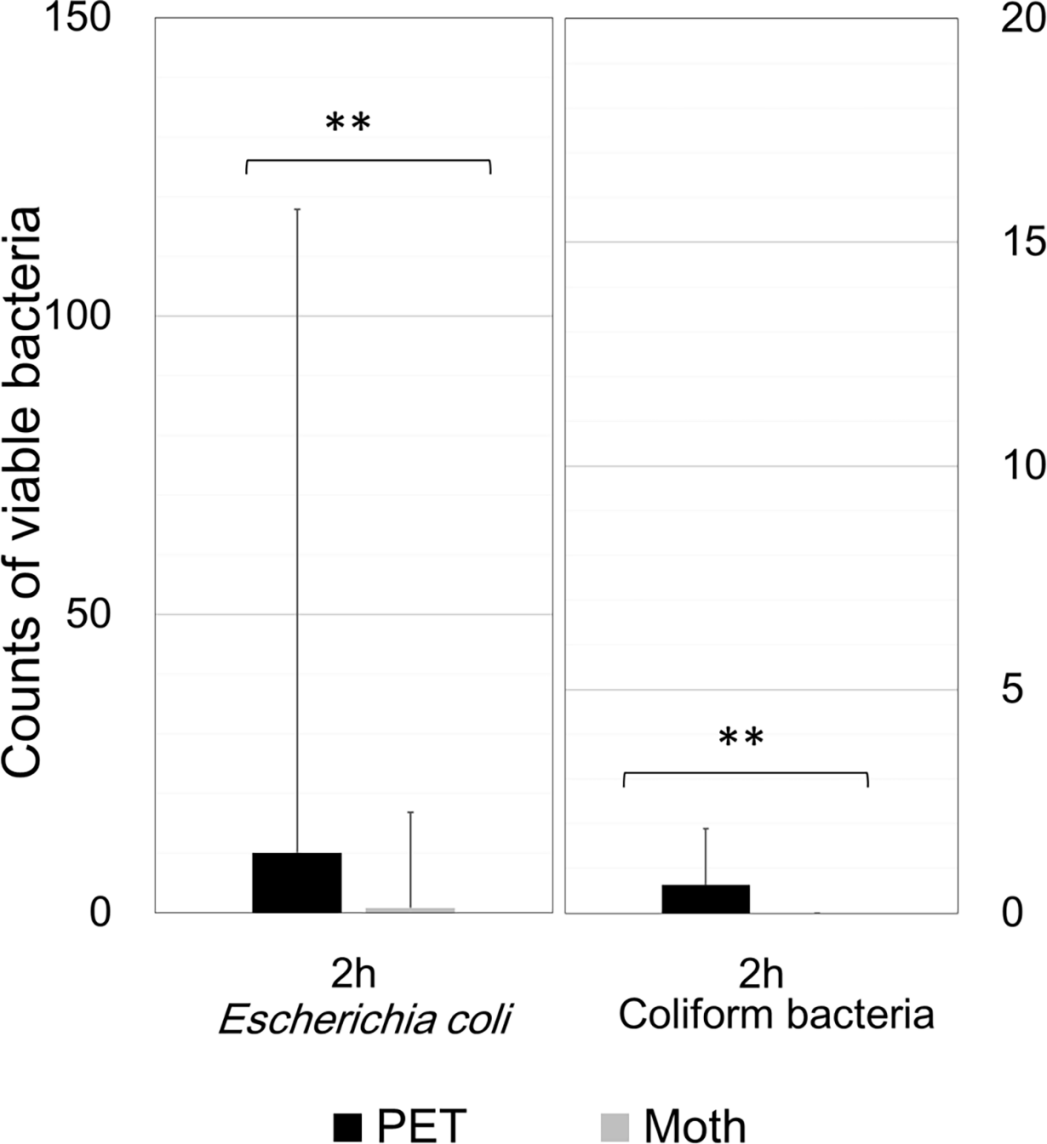
*Escherichia coli* and coliform bacteria counts on the ESCM stampers at the company cafeteria. Average numbers of viable bacteria, *Escherichia coli* (a) or coliform bacteria (b), collected from the Moth-eye and PET films at two hours. ** p<0.05, Mann-Whitney test. n = 46.

However, for both *E*. *coli* and coliform bacteria, the average counts from the Moth-eye film were nominally lower than those from the PET film, but the differences were not significant, possibly due to the large variations. To collect more samples, we carried out the study at the company cafeteria and the results are shown in Fig 5. For both *E*. *coli* and coliform bacteria, the mean counts collected from the Moth-eye film were significantly lower than those from the PET film (p<0.05). These results suggested that the Moth-eye film might be useful for inhibiting the growth of bacteria such as *S*. *aureus* and *E*. *coli*, which are species associated with highly pathogenic hospital infection.

**Fig 6:**
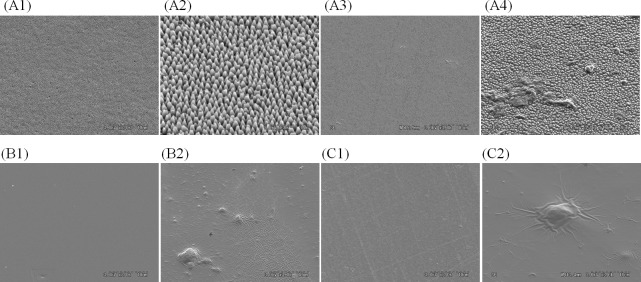
Representative SEM micrographs of prepared surfaces and those collected from the basin. (A1) and (A2) prepared Moth-eye film, (B1) prepared Flat film, (C1) prepared PET film, (A3) and (A4) Moth-eye film collected from the basin at 16:00, (B2) Flat film collected from the basin at 16:00 and (C2) PET film collected from the basin at 16:00. Magnifications are x3000 for A1, A3, B1, B2, C1 and C2, x10,000 for A4, x30,000 for A2. All images were taken at 3 kV.

Representative SEM micrographs of prepared surfaces and those collected from the basin are shown in Fig 6. As can be seen in Fig 6 (A2), the height and the pitch of the Moth-eye nanostructures used in this study were approximately 200 nm. After testing, a small amount of adhered material was observed on all of the Moth-eye, Flat and PET films. Part of the adhered material was considered to be single bacteria, but no colony-forming bacteria were observed. Compared to the Flat (Fig 6 (B2)) and PET films (Fig 6 (C2)), the Moth-eye film showed a smaller amount of adhered material on its surface (Fig 6 (A3)). The thickness of the adhered material on surface of the Moth-eye film was a few micrometers at most (Fig 6 (A4)).

In the meantime, uniquely shaped material was found on the surface of the Flat and PET films. As shown in Fig 6 (B2, C2), the material formed a wrinkle-like pattern of several tens to hundreds of micrometers in length, surrounding the nucleus of spherical bacteria. The surfaces of the Flat and PET films were covered with this wrinkled material. These results indicated that the adhered material on the Flat and PET films was likely to be thicker than that on the Moth-eye film.

On the assumption that the thick layer of adhered wrinkle-shaped material was composed of biological secretions such as proteins, the SEM observation results mentioned above could explain the difference among the antibacterial effects of the Moth-eye, Flat and PET films as follows. The smaller amount of adhered material on the Moth-eye film indicates a smaller amount of biological secretions (protein) and, as a result, the bacteria growth can be prevented to a greater degree on the surface of the Moth-eye film in comparison to that on the Flat and PET films. In addition, bacterial biofilm formation can be inhibited by the combined effect of the reduced adhesion and reduced colony formation observed in our previous *in vitro* study [20].

In this study, an insufficient number of typical spherical or rod-like bacteria and bacterial colonies were observed because of the limited testing time. Further, detailed principle component analysis of the thick layer of adhered wrinkle-shaped material found on the Flat and PET films has not yet been completed at this moment. A further study on the antibacterial efficacy of the Moth-eye film in practical settings should be conducted.

Based on the results of our previous study, which showed a difference in the formation of colonies between nanostructured and flat surfaces, further studies from a microscopic perspective in a practical setting should also be carried out to examine whether or not biofilms, for example, form.

## Conclusions

This study found that Moth-eye film applied in public facilities had an antibacterial effect that appeared to depend on both its physical (nanostructure) and chemical (hydrophilic resin) properties. The safety of the hydrophilic resin-coated PET film was previously confirmed to have no impact on rabbit skin by Animal Irritation test. The advantages of the Moth-eye film include this material’s ability to bring about a decrease in contact infection risk due to its immediate antibacterial activity, along with longer-term suppression of bacterial growth. The Moth-eye film appears to be applicable to any equipment or surface in close proximity to water, such as hand dryers, bathroom door knobs, water condensation trays, and so on. Further studies to investigate the efficacy of this film for periods over 6 hours under various conditions are needed to demonstrate the broad applicability of the Moth-eye film in such settings.
